# Preparation of Bromide of Iron

**Published:** 1844-12-14

**Authors:** 


### PREPARATION OF BROMIDE OF IRON.

Iron filings, thirty grammes. This substance is introduced into a glass flask, which can be hermetically sealed; then there is added ninety to 100 grammes of distilled water; then there is ten grammes of bromine gradually poured on the whole. The flask is carefully corked, and agitated, from time to time, until the liquor takes a greenish tint; after which the product is filtered, and promptly evaporated until dry. The bromide of iron has a brick-red colour; it is deliquescent, readily dissolves in water, aud has a very styptic taste.—*Pharmacopœia Universalis of Geiger and Mohr, and Chemist.*

